# A Review on Structure, Modifications and Structure-Activity Relation of Quercetin and Its Derivatives

**DOI:** 10.4014/jmb.1907.07003

**Published:** 2019-11-11

**Authors:** Rubin Thapa Magar, Jae Kyung Sohng

**Affiliations:** 1 Department of Life Science and Biochemical Engineering, SunMoon University, Asan 3460, Republic of Korea; 2 Department of Pharmaceutical Engineering and Biotechnology, SunMoon University, Asan 31460, Republic of Korea

**Keywords:** Quercetin, glycosylation, methylation, biotransformation

## Abstract

Quercetin and its derivatives are important metabolites that belong to the flavonol class of flavonoids. Quercetin and some of the conjugates have been approved by the FDA for human use. They are widely distributed among plants and have various biological activities, such as being anticancer, antiviral, and antioxidant. Hence, the biosynthesis of novel derivatives is an important field of research. Glycosylation and methylation are two important modification strategies that have long been used and have resulted in many novel metabolites that are not present in natural sources. A strategy for modifying quercetin in *E. coli* by means of glycosylation, for example, involves overexpressing respective glycosyltransferases (GTs) in the host and metabolic engineering for increasing nucleoside diphosphate sugar (NDP-sugar). Still others have used microorganisms other than *E. coli*, such as *Streptomyces* sp., for the biotransformation process. The overall study of the structural activity relationship has revealed that modification of some residues in quercetin decreased one activity but increased others. This review summarizes all of the information mentioned above.

## Introduction

Quercetin is one of the important flavonoids that belongs to the flavonol class of flavonoids and are distributed widely among the fruits and vegetables, such as onion [1] and mango [2]. Plants contain not only aglycone (quercetin) but also the various types of conjugated forms of quercetin with glycosides as well as methyl ethers. One of the important derivatives, isoquercetin, also known as isoquercetrin or isotrifolin is found to be present in onions [1] and mangoes [2]. Other important derivatives includes rutin which is available mainly in citrus foods.

Quercetin and its derivatives are pharmacologically active compounds, because they show various biological activities. Quercetin has an anticancer activity as an inhibitor of human cathepsin B [3]. Many others derivatives have antiviral activities; for example, quercetin 3-*O*-β-D-glucoside (Isoquercetrin) works against ebola virus [4]. There are other methylated derivatives that have different activities. As for example, rhamnetin (7-*O*-methyl quercetin) has an anti-inflammatory effect [5].

Enzymatically modified isoquercitrin (EMIQ) is another major quercetin derivatives with significant applications. It is more beneficial than rutin because it is highly water soluble and more easily absorbed than other quercetin glycosides and quercetin itself [6]. It has been granted approval as a food additive in Japan and is generally regarded as safe (GRAS) safe as an antioxidant by the U.S. FDA. It has been used as a dietary supplement as bioactive quercetin.

## Structure of Quercetin and Its Derivatives

Quercetin (2-(3,4-dihydroxyphenyl)-3,5,7-trihydroxy-4H-chromen-4-one), a major class of flavonoids, contains five hydroxyl groups at 3,5,7,3’ and 4’ of the basic skeleton of flavonol. Some of these hydroxyl groups are glycosylated to various quercetin glycosides and constitute the major quercetin derivatives.

Structurally, isoquercetin contains glucose attached to the 3-OH group of quercetin. Moreover, galactose attachment to the 3-OH of quercetin leads to the synthesis of hyperoside (quercetin 3-*O*-galactoside). Similarly, the rhamnosyl group also attaches to 3-OH or 7-OH and leads to the synthesis of quercetin 3-*O*-rhamnoside and quercetin 7-*O*-rhamnoside, respectively.

Some quercetin derivatives also contain disaccharides, such as rutinose, which is composed of rhamnose and glucose group and is named α-L-rhamnopyranosyl-(1 → 6)-β-D-glucopyranose. Attachment of this disaccharide at the 3-OH position of quercetin leads to the important compound named rutin. Likewise, avicularin contains the arabinofuranose attached to quercetin 3-OH.

Some quercetin derivatives have more than two sugar residues as well. Examples include enzymatically modified isoquercetrin (EMIQ) and oligoglucosylated rutin. The former contains up to 10 glucose residues attached to the 3-OH of quercetin, whereas the latter contain up to five more glucose residues attached to the glucose residue of rutin.

There are also some methylated quercetin derivatives. For example, tamrixetin, quercetin 4’-methyl ether, contains an extra methyl group at the 4’ position of quercetin. Similarly, another methylated quercetin, rhamnetin 7-*O*-methyl quercetin has a methyl group at 7-OH of quercetin. Moreover, dimethylated quercetin, named rhamnazin, contains two methyl groups at the 3’- and 7-OH groups of quercetin. Isorhamnetin is another methylated flavonol, also known as 3-methylquercetin and isorhamnetol. It can be glycosylated to form isorhamnetin 3-*O*-rutinoside (narcissin), isorhamnetin 3-*O*-rutinoside-7-*O*-glucoside, and isorhamnetin 3-*O*-rutinoside-4’-*O*-glucoside.

**Fig. 1:**
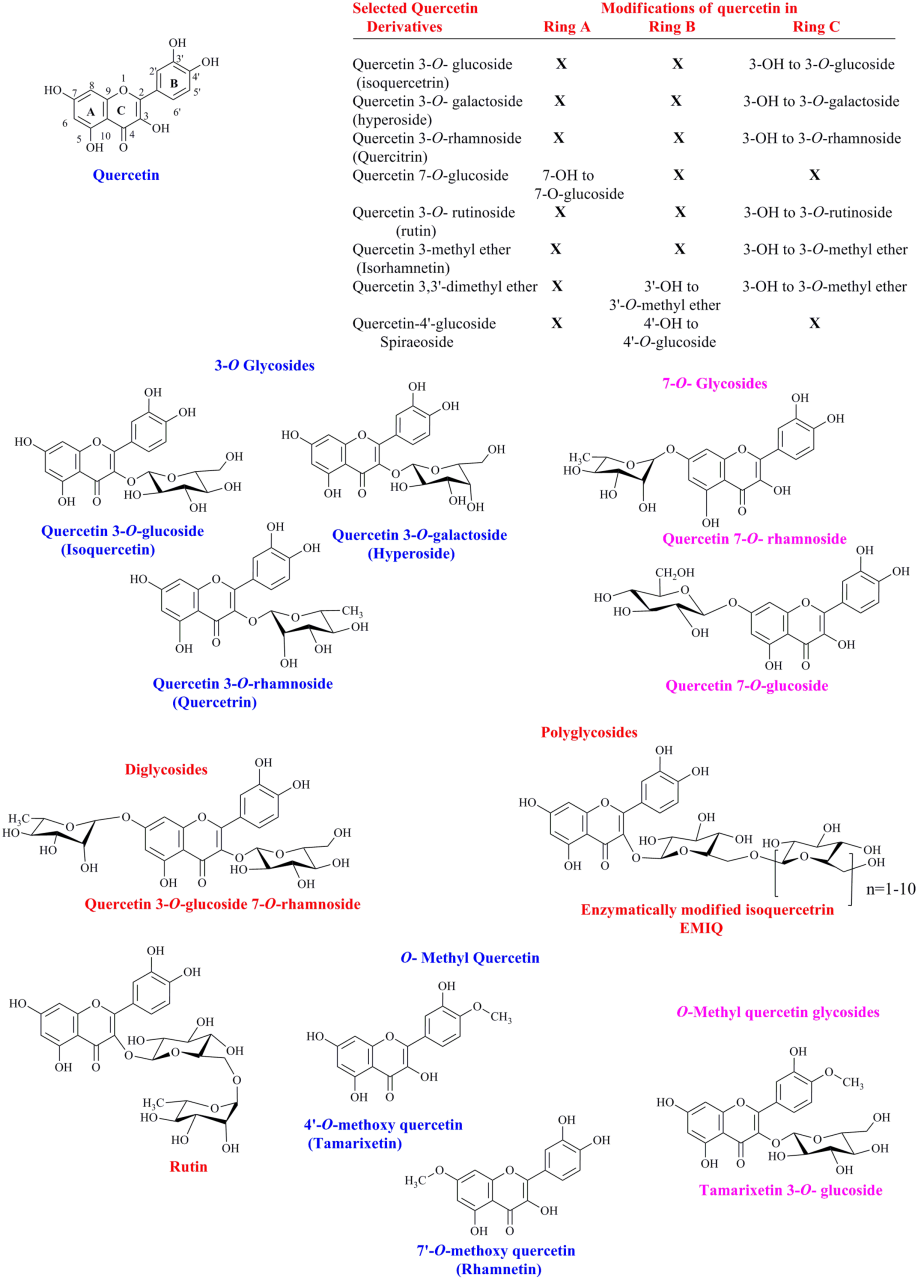
Structure of quercetin and some of its derivatives.

There are more structural diversities in quercetin that contains both the glycosyl group and methyl groups attached to OH groups of quercetin. For example, tamarixetin 3-*O*-*β*-D-glucoside contains a methyl group at the 4’ position and glucose at the 3 position. Structures of some of the selected quercetin conjugates are given in Fig. 1.

## Approaches for Quercetin Modifications

Quercetin derivatives have various biological activities. Hence, the modification of quercetin has been extensively studied in the field of flavonoids research in order to generate various quercetin derivatives with diverse structures and functions. Biotransformation is an important tool for the modification of quercetin, where the microorganisms are engineered to express the desired biocatalysts that do the desired modification, such as glycosylations or methylations. For these desired modification processes, most researchers use metabolically engineered *E. coli* strain, and others use microorganisms such as *Streptomyces* sps. Moreover, in vitro modification with purified biocatalysts is another tool for synthesis of quercetin derivatives.

## In Vitro Modification of Quercetin

**Fig. 2:**
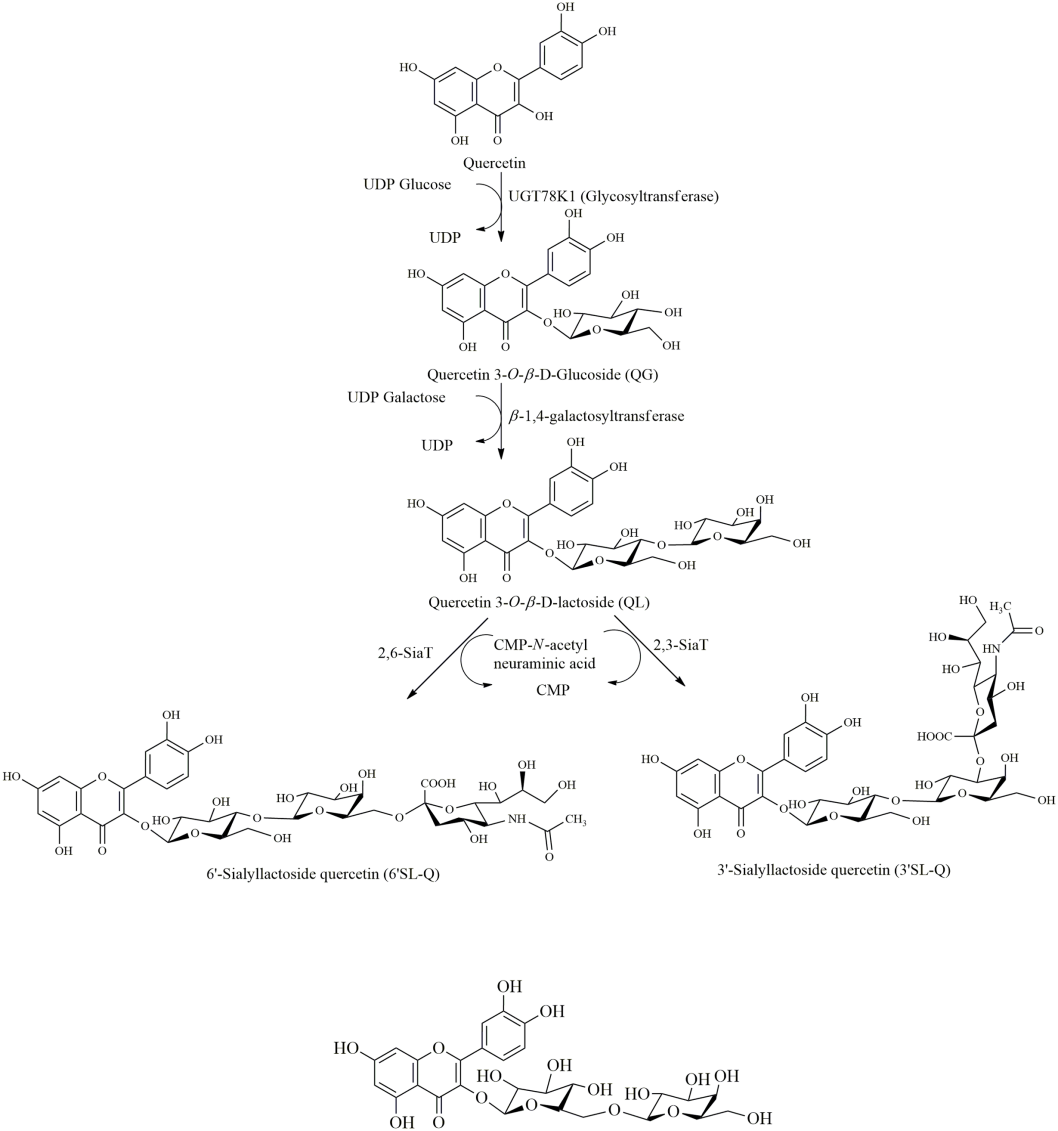
Biosynthesis of quercetin sialyllactoside derivatives. Two step reactions catalyzed by two glycosyltransferases (UGT78K1 and β-(1,4)-GalT) separately and sequentially produce quercetin 3-*O*-Dlactoside (QL) which then under the action of two sialyltransferase (2,3-SiaT and 2,6-SiaT) produce 3’-sialyllactosyl quercetin (3’-SL-Q) and 6’- sialyllactosyl quercetin (6’-SL-Q) respectively.

**Glycosylation.** There are various instances where the purified biocatalysts are used for the modification of quercetin. All the biocatalysts are overexpressed, purified and used for the modification of quercetin. In one example, the in vitro synthesis of two novel quercetin sialyllactoside derivatives, quercetin-3-*O*-β-D-glucopyranosyl, 4’’-*O*-D-galactopyranosyl 3’’’-*O*-α-N-acetyl neuraminic acid, *i.e.*, 3’-sialyllactosyl quercetin (3’-SL-Q) and quercetin-3-*O*-β-D-glucopyranosyl, 4’’-*O*-β-D-galactopyranosyl 6’’’-*O*-α-N-acetylneuraminic acid, *i.e.*, 6’-sialyllactosyl quercetin (6’-SL-Q) was performed [7]. They used glucosyltransferase UGT78K1 (*Glycine max*), β-1,4-galactosyltransferase (*Helicobacter pylori*), and two different sialyltransferases, α2,3-SiaT and α2,6-SiaT. At first, they synthesize the quercetin-3-*O*-β-D-glucoside (QG) by using a one-pot reaction system having a crude enzyme UMK (UMP kinase), ACK (acetate kinase), GalU (UDP-α-D-glucose synthase), and UGT78K1 (glycosyltransferase). More than 90% conversion was seen. Then, they converted QG to quercetin 3-*O*-β-D-lactoside (QL) by using QG as the substrate and β-1,4-galactosyltransferase as GT to achieve more than 95%conversion. Finally, the desired products were formed by using QL as the substrate and α2,3-SiaT and α2,6-SiaT as sialyltransferases. The conversion rate was almost 95%. This is summarized in Fig. 2.

**Production of EMIQ from rutin.** It is an important method for the modification of rutin to important derivatives like EMIQ (enzyme modified isoquercetrin). Unlike all other methods described above, glycosidase acts as the most important enzyme in this modification strategy.

*Rutin to isoquercetrin* (*IQ*): Isoquercetrin is an important quercetin derivative, because it is the substrate for another important quercetin derivative known as EMIQ and has various biological activities as well. Several methods have been used for the synthesis of this compounds. α-L-rhamnosidases is an enzyme that can hydrolyze the rhamnosyl group of rutin to produce isoquercetrin. There are several pieces of evidence of isoquercetrin production from rutin using rhamnosidase enzymes. In one evidence, it was produced by using the naringinase (EC 3.2.1.40) from *Penicillium decumbens* [8]. In this case, a maximum conversion was found to be 95.20 ± 2.52% and is more economical than is the conventional method.

Other enzymes, herperidinase from *Penicillum* sp. and naringinase from *Penicillium decumbens*, were used for the conversion of rutin to IQ by inactivating the undesirable β-D-glucosidase activity by heating to 70°C for 30 min [9].

*Rutin to EMIQ*: EMIQ is produced from rutin via a two-step reaction. At first step, α-L-rhamnosidases activity leads to the derhamnosylation of rutin to produce isoquercetrin. Next step produces the enzymatically modified isoquercetrin under the action of cyclodextrin glucanotransferase (CGTase) [10]. Another enzyme, amylosucrase (ASase) from *Deinococcus geothermalis*, was also used by to convert isoquercetrin to EMIQ [11]. This enzyme uses sucrose rather than dextrin as co- substrate. They found the conversion rate of 97% for ASase as compared to that of CGTase of only 75% (Table 1).

**Fig. 3:**
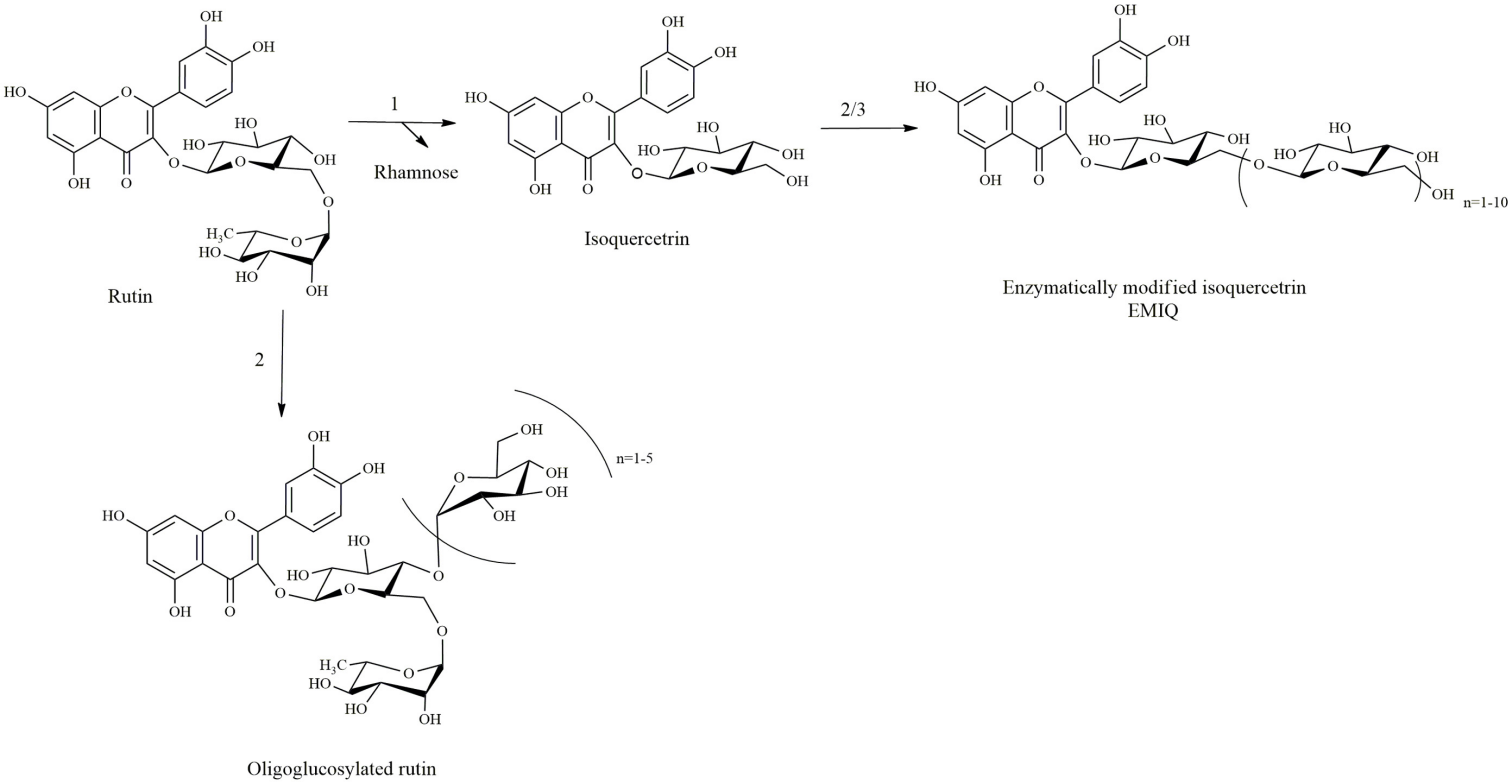
Conversion of rutin to EMIQ. Rhamnosidase releases the rhamnose from rutin and under the action of cyclodextrin glucanotransferase or amylosucrase converts to EMIQ. Cyclodextrin glucanotransferase catalyze the synthesis of oligoglucosylated rutin without action of rhamnosidase [1. Rhamnosidase, 2. Cyclodextrin glucanotransferase (dextrin as cosubstrate), 3. Amylosucrase (sucrose as cosubstrate)].

The transglycosylation of rutin is another type of modification, where rutin is oligoglycosylated without hydrolyzing the rhamnose sugar. As a result, oligo-saccharides are attached to the glucoside group of rutin. The enzyme CGTase can do this with dextrin as a sugar group donor. The overall biosynthetic steps are summarized in Fig. 3.

## Metabolic Engineering of *E. coli* for Modification of Quercetin

**Fig. 4:**
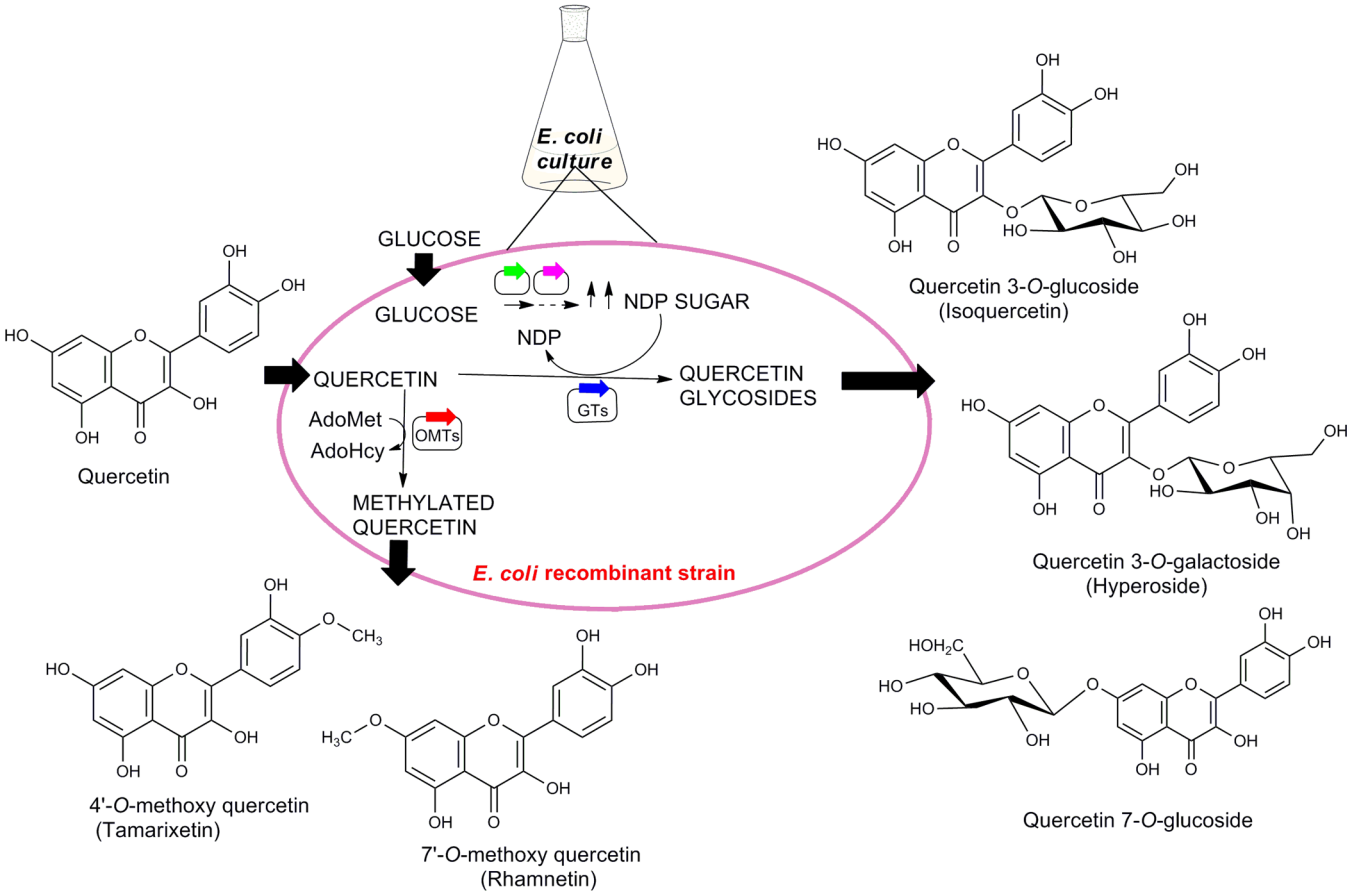
Biotransformation in engineered *E. coli*. Engineered *E. coli* produce glycosyltransferases (GTs) and nucleoside diphosphate sugar (NDP-sugar). The NDP-sugar is used by GTs for the formation of quercetin glycosides. On the other hand, O-methyl transferase (OMTs) uses S-adenosylmethionine (AdoMet) for methylation of quercetin releasing S-adenosylhomocysteine (AdoHcy).

*E. coli* is engineered to express the desired biocatalysts isolated from various sources and deletion of unnecessary genes and overexpression of some other genes of *E. coli* are also performed in some cases. Then, this engineered *E. coli* is used for biotransformation of quercetin. The brief overview of this strategy is figuratively described in Fig. 4.

**Glycosylation.** Glycosylation of quercetin is the most studied modification strategy in the field of producing glycosides derivatives. The glycosyltransferases (GTs) are biocatalysts that are overexpressed in *E. coli* that glycosylates quercetin to produce desired modification. The biosynthesis quercetin 3-*O*-glucoside is one of the few examples where *E. coli* is engineered to overexpress RF5 (Flavanol-3-*O*-glucosyltransferse) [12]. Here, the source of the former enzyme is *Oryza sativa* while for the latter is *Bacillus cereus* to enzyme is *Oryza sativa*.

In some cases, the required nucleoside diphosphate sugar (NDP sugar) is not synthesized in *E. coli*. Hence, in this case, respective genes for their synthesis and the required glycosyltransferase are overexpressed together. As a result, a novel quercetin glycoside with a different sugar is synthesized. One of the examples is a novel quercetin glycosides (quercetin 3-*O*-(6-deoxytalose) which was synthesized by using genes *tll* (encodes dTDP-6-deoxy-L-lyxo-4-hexulose reductase) from *Actinobacillus actinomycetemcomitans* that convert dTDP-4-dehydro-6-deoxy-L-mannose to dTDP-6-deoxytalose, and glycosyl transferase *AtUGT78D1* (ﬂavonol 3-*O*-rhamnosyltransferase) from *Arabidopsis thaliana* that produces quercetin 3-*O*-6-deoxytalose [14]. Moreover, they also engineered the nucleoside diphosphate biosynthetic genes in *E. coli* for increased synthesis of dTDP-6-deoxytalose. They deleted some genes of the dTDP-L-rhamnose biosynthetic pathway, such as galU (UTP-glucose 1-phosphate uridyltransferase), *rffA* (dTDP-4-oxo-6-deoxy-D-glucose transaminase), *rfbD* (dTDP-4-dehydrorahmnose reductase), and overexpressed *tll* *AtUGT78D1*. This synthesized mutant produced > 6.5-fold higher quercetin 3-*O*-(6-deoxytalose) than did the wild type.

In other case, two glycosyltransferases are overexpressed in *E. coli* in order to generate the quercetin diglycosides. Example includes rutin which was synthesized at a concentration of about 119.8 mg/l from engineered *E. coli* in such a way that one UGT *BcGT1* from *Bacillus cereus* was integrated into an *E. coli* chromosome, and other UGT (UGT, *Fg2* from G. max) was expressed along with the rhamnose synthase gene (*RHM2*) [15]. Hence by optimization of the steps, they produced rutin from quercetin in a significant amount.

Similarly, a stepwise method for synthesis of quercetin diglycosides was also performed [16]. At first, they synthesized quercetin-3-*O*-glucoside (Q-G) and quercetin 3-*O*-arabinose (Q-A) from the engineered *E. coli* that contained genes for glycosyltransferase as well as for UDP-glucuronic acid [17] and the UDP-arabinose synthetic pathway [18]. The next step was conversion to quercetin diglycosides, quercetin 3-*O*-glucuronic acid 7-*O*-rhamnoside [Q-GR] and quercetin 3-*O*-arabinose 7-*O*-rhamnoside [Q-AR] by using a strain harboring AtUGT89C1 and the rhamnose synthase gene RHM. In this way, they found the conversion rate to be 77.7% and 71.8% for Q-AR and Q-GR, respectively, starting from quercetin.

More recently, a synthetic approach for the biosynthesis of another derivatives of quercetin, miquelianin (Quercetin 3-*O*-glucuronide) was designed [19]. Quercetin was converted to miquelianin by the engineered *E. coli* strain harboring the genes for UDP-glucuronic acid biosynthesis along with glucokinase and grapevine (*Vitis vinifera*) glycosyltransferase *VvGT5* (UDP-glucuronic acid: flavonol 3-*O*-glucuronosyltransferase). As a result, the conversion of quercetin to miquelianin was found to be 31% and production was 30 mg/l (62 μmol/l).

This is how desired glycosylated derivatives of quercetin are produced in engineered *E. coli*. Table 2 summarizes the biosynthesis of some of the important quercetin glycosides and the respective glycosyltransferase used.

**Methylation.** Methylation is another important modi-fication of quercetin. O-methyl transferases (OMTs) catalyzes the methylation of quercetin in presence of S-adenosylmethionine (AdoMet) which acts as methyl donor producing methylated quercetin and S-adenosylhomocysteine (AdoHcy) the methylation of quercetin. Quercetin was modified to 3’-methylated and 3’, 4’-dimethylated quercetin using two methyltransferase ROMT-9 and SOMT2 from Rice and *Glycine max* respectively by overexpression in *E. coli* [20]. They found that more than 90% of quercetin was converted to dimethylated quercetin. Moreover, methyl transferases from bacterial origin, such as GerMIII from *Streptomyces* sp. KCTC 0041BP [21], has been used for the synthesis of 4’-*O*-methyl-quercetin (Table 3).

## Quercetin Modification with Other Native Microorganisms

Some other microorganisms besides *E. coli* has been using for the modification of quercetin. Here, these microorganisms are not engineered to overexpress the glycosyltransferase and respective NDP-sugar biosynthesis gene. Instead, the wild type strain as such is used for modifications where the native enzymes do the desired biological reactions.

**Glycosylation.** Besides *E. coli*, other microorganisms significantly do the glycosylation of quercetin. An important derivative, isoquercetrin (quercetin-3-*O*-β-D-glucopyranoside), was produced in *Gliocladium deliquescens* NRRL 1086 after the bioconversion of quercetin [22]. Interestingly, microbial glucuronidation is another method for the generation of quercetin glycosides. Biotransformation of quercetin in the *Streptomyces* strain M52104 resulted in many glucuronides derivatives, such as quercetin 4’-*O*-β-glucuronide (50%), quercetin 3-*O*-*β*-glucuronide, quercetin 7-*O*-β-glucuronide (together 38%), and quercetin 3’-*O*-*β*-glucuronide [23]. A novel quercetin glycoside, quercetin-7-*O*-*β*-4’’-deoxy-hex-4’’-enopyranosiduronic acid, was produced by biotrans-formation of quercetin in *Streptomyces rimosus* subsp. *rimosus* ATCC10970. The complete conversion was seen within three days of culture [24]. These are summarized in Table 4.

**Methylation.** Like glycosylation, different microorganisms have been used for synthesis of methylated quercetin. To name a few, *Streptomyces griseus* ATCC13273 was used for quercetin biotransformation which resulted in mono- and dimethoxy ring B metabolites 3’-*O*-methyl quercetin, and 3,5,7-trihydroxy-3’,4’-dimethoxyflavone [25]. *B. bassiana* ATCC7159 was used for 3’-*O*-methylquercetin and 3’-*O*-methylquercetin-7-glucuronide metabolites [26]. These are all summarized in Table 5.

**Hydroxylation and sulfation.** The modification of quercetin with a hydroxyl group has been studied less than glycosylation and methylation. In one study, myricetin (3,5,7, 3’,4’, 5’-hexahydroxyflavone) was produced by *Streptomyces griseus*, and the same strain also produced the plant flavonoid gossypetin (3,5,7,8,3’,4’-hexahydroxyflavone) after biotransformation of quercetin that showed the 4’-and 8-hydroxylation [25].

Sulfation is another type of modification found in quercetin. In one of the works, quercetin was successfully sulfated to quercetin 3’-*O*-sulfate via the biotransformation in *Beauveria bassiana* ATCC7159 [26]. They checked the biotransformation of quercetin not only in *B. bassiana* ATCC7159 but also in 10 isolates of *Beauveria sp* strains isolated from soil samples of central Brazil. All of the strains successfully produced the sulfated quercetin.

## Structural Activity Relationship of Quercetin and Its Derivatives

Studies of the biological activities of quercetin and its derivatives have concluded that they have different activities and efficacies because of modification at important positions of the quercetin molecule. The modification with glycosides usually occurs at the 3-OH and 7-OH positions, whereas methylations is usually preferred at the 3’, 4’ and 7-OH positions. In one of the works, the structure activity relationship of quercetin and its derivatives on antioxidant and anti-inflammatory activities was studied [27]. They have identified that modification of quercetin reduces its antioxidant activity. They found the overall activity as quercetin > tamarixeti*n* = isorhamnetin > quercetin-3-*O*-glucuronide>isorhamnetin-3-*O*-glucoside>quercetin-3,5,7,3’,4’-penthamethylether > quercetin-3,4’-di-*O*-glucoside. These data said that the 3-hydroxyl group of quercetin plays an important role for antioxidant activity, and the mechanism was already described [28]. Similarly, the inhibition of lipid peroxidation was also studied, and the methylated quercetin metabolites tamarixetin and isorhamnetin have higher activity than does quercetin [27]. Their results were completely correlated with the study carried out previously [29].

The anti-inflammatory activity of monomethylated quercetin tamarixetin was highest among quercetin and its metabolites [27]. Hence they concluded that, unlike the antioxidant activity, the anti-inflammatory activity is not completely dependent on the number of free hydroxyl groups.

The anti-obesity effects of quercetin and quercetin 3-glycosides have been studied and found that the latter has a higher effect than does the former [30], perhaps because better bioavailability results in better bioactivity, since the bioavailability of quercetin 3-O glycosides is 235% higher than the 100% for quercetin [30, 28].

In conclusion, quercetin and its derivatives are important metabolites having various activities and efficacies. Moreover, a particular metabolite can have high biological activity and can be used pharmacologically. Hence for generation of active metabolites, modification of quercetin should be done. The important reactions are glycosylation, methylations, and hydroxylations. Glycosylation produces quercetin derivative with various sugar units at different hydroxyl groups, which creates not only the novel metabolites but also the increased activity and functions different from those of the parent. Hence, finding biocatalysts that could modify a parent molecule to novel analogues is of great interest to many researchers. Likewise, study of the structural activity relationship of quercetin and its derivatives also provides information for increasing the efficacy of the metabolites with decreasing toxicity. There is still some work to be done for understanding the change in activity of metabolites with the change in structure. This could provide an important field of research to some researchers as well.

## Figures and Tables

**Table 1:** Some selected in vitro modification of quercetin.

Quercetin conjugates	Biocatalysts used	Sources/activities of biocatalyst	References
3’-Sialyllactosyl quercetin (3’-SL-Q) and 6’-sialyllactosyl quercetin (6’-SL-Q)	UGT78K1 (glycosyltransferase) β-1,4-galactosyltransferase	*Glycine max*/quercetin to quercetin 3-*O*-β-D- glucoside *Helicobacter pylori*/ conversion of quercetin 3-*O*-β-D- glucoside to quercetin 3-*O*- β-D-lactoside	7
	Sialyltransferases, α2,3-SiaT and α2,6-SiaT	Pasteurella multocida/ quercetin 3-*O*- β-D-lactoside to 3’-sialyllactosyl quercetin (3’-SL-Q) and 6’-sialyllactosyl quercetin (6’-SL-Q) respectively	
Isoquercetrin (IQ) from rutin	Naringinase	*Penicillium decumbens*/ derhamnosylation of rutin	8, 9
	Hesperidinase	*Penicillium* sp./ derhamnosylation of rutin	9
EMIQ from Isoquercetrin	Cyclodextrin glucosyltransferase (CGTase)	*Bacillus macerans*/ oligoglucosylation of IQ	10
	Amylosucrase (ASase)	*Deinococcus geothermalis*/ oligoglucosylation of IQ	11

**Table 2:** Some selected glycosylation of quercetin in *E. coli*.

Quercetin glycosides	Biocatalysts used in *E. coli*	Sources/activities of biocatalyst	References
Quercetin 3-*O*-glucoside	RF5 (Flavanol-3-*O*-glucosyltransferse)	*Oryza sativa*/ glycosylation of quercetin	12
Quercetin 3-*O*-xyloside	Phosphoglucomutase (nfa44530)	*Nocardia farcinica*/ glucose 6 phosphate to glucose 1 phosphate	13
	Glucose-1-phosphate uridylyltransferase (*galU*).	*E. coli* *K-12*/glucose 1 phosphate to UDP glucose	
	UDP-glucose dehydrogenase (*calS8*) and UDP-glucuronic acid decarboxylase	*Micromonospora echinospora* spp. *Calichensis*/UDP glucose to UDP xylose	
	*arGt-3* (*Glycosyltransferase*)	*Arabidopsis thaliana*/quercetin to quercetin 3-*O*-xyloside.	
Quercetin 3-*O*-(6-deoxytalose)	*tll gene* (encoding dTDP-6-deoxy-l-lyxo-4-hexulose reductase)	*Actinobacillus actinomycetemcomitans* /Convert dTDP-4-dehydro-6-deoxy-l-mannose to dTDP-6-deoxytalose.	14
	*AtUGT78D1* (ﬂavonol 3-*O*-rhamnosyltransferase)	*Arabidopsis thaliana*/Quercetin to quercetin 3-*O*-(6-deoxytalose).	
Quercetin 3-*O*-glucosyl (1→2) xyloside	AtUGT78D2 and AtUGT79B1	*Arabidopsis thaliana*/ quercetin to quercetin 3-*O*-glucoside and then to quercetin 3-*O*-glucosyl (1→2) xyloside.	15
Quercetin 3-*O*-glucosyl (1→6) rhamnoside (Rutin)	AtUGT78D2 and Fg2.	*Arabidopsis thaliana*/ quercetin to quercetin 3-*O*-glucoside and *G. max*/to rutin.	
Quercetin 3-*O*- glucuronic acid 7-*O*- rhamnoside [Q-GR] and quercetin 3-*O*- arabinose 7-*O*-rhamnoside [Q-AR]	AtUGT89C1 and rhamnose synthase gene RHM (Rhamnose synthase)	*Arabidopsis thaliana*/rhamnosylation of quercetin	16
			17
			18
Miquelianin (Quercetin 3-glucuronide)	UDP-glucuronic acid biosynthesis genes along with glucokinase		19
	Glycosyltransferase *VvGT5* (UDP-glucuronic acid: flavonol 3-O glucuronosyltransferase)	Grapevine (*Vitis vinifera*)/Quercetin to quercetin 3-glucuronide	

**Table 3:** Some selected methylation of quercetin in *E. coli*.

Methylated quercetin	Biocatalysts used in *E. coli*	Sources/activities of biocatalyst	References
3’-Methylated and the 3’,4’-dimethylated quercetin	ROMT-9 and SOMT2	Rice and *Glycine max*/methylates 3’ and 4’ OH of quercetin	20
4’-*O*-Methyl-quercetin	GerMIII	*Streptomyces* sp. KCTC 0041BP/methylates at 4’ OH of quercetin	21

**Table 4:** Some selected glycosylation of quercetin in other native bacterial strains.

Quercetin glycosides	Microorganisms used for biotransformation	References
Quercetin 3-*O*-β-D-glycoside	*Gliocladium deliquescens*NRRL 1086/quercetin to quercetin glycoside	22
Quercetin 4’-*O*-β-glucuronide	*Streptomyces* M52104/quercetin to quercetin glucuronides	23
Quercetin 3-*O*-β-glucuronide		
Quercetin 7-*O*-β-glucuronide		
Quercetin 3’-*O*-β-glucuronide		
Quercetin-7-*O*- β-4’’-deoxy-hex-4’’-enopyranosiduronic acid	*Streptomyces rimosus* subsp. rimosus ATCC 10970/quercetin to Quercetin-7-*O*- β-4’’-deoxy-hex-4’’-enopyranosiduronic acid	24

**Table 5:** Some selected methylation of quercetin in other native bacterial strains.

Methylated quercetin	Microorganisms used for biotransformation	References
3’-*O*-Methyl quercetin,	*Streptomyces griseus*/quercetin to methylated conjugates	25
3,5,7-Trihydroxy-3’,4’-dimethoxyflavone		
3,5,7,3’,4’-Pentahydroxy-8-methoxyflavone (8-methoxyquercetin)		
3’-*O*-Methylquercetin	*B. bassiana* ATCC 7159/quercetin to methylated quercetin and methylated quercetin glucuronides	26
3’-*O*-Methylquercetin-7-glucuronide		
